# Boosted Convolutional Neural Network Algorithm for the Classification of the Bearing Fault form 1-D Raw Sensor Data

**DOI:** 10.3390/s23094295

**Published:** 2023-04-26

**Authors:** Paweł Knap, Krzysztof Lalik, Patryk Bałazy

**Affiliations:** Faculty of Mechanical Engineering and Robotics, AGH University of Science and Technology, al. Adama Mickiewicza 30, 30-059 Cracow, Poland; pknap@agh.edu.pl (P.K.);

**Keywords:** vibrodiagnostics, neural networks, predictive maintenance, structural health monitoring, bearing fault detection

## Abstract

Renewable energy sources are a growing branch of industry. One such source is wind farms, which have significantly increased their number over recent years. Alongside the increased number of turbines, maintenance problems are growing. There is a need for newer and less intrusive predictive maintenance methods. About 40% of all turbine failures are due to bearing failure. This paper presents a modified neural direct classifier method using raw accelerometer measurements as input. This proprietary platform allows for better damage prediction results than convolutional networks in vibration spectrum image analysis. It operates in real time and without signal processing methods converting the signal to a time–frequency spectrogram. Image processing methods can extract features from a set of preset features and based on their importance. The proposed method is not based on feature extraction from image data but on automatically finding a set of features from raw tabular data. This fact significantly reduces the computational cost of detection and improves the failure detection accuracy compared to the classical methods. The model achieved a precision of 99.32% on the validation set, and 96.3% during bench testing. These results were an improvement over the method that classifies time–frequency spectrograms of 97.76% for the validation set and 90.8% for the real-world tests, respectively.

## 1. Introduction

Mechanical vibrations are a common phenomenon occurring all around us. Unfortunately, their influence can often be destructive. Industrial machines and their parts are particularly exposed to the harmful effects of vibrations. Bearings are one of these parts. Their proper examination is crucial for the safe functioning of machines. Unfortunately, the traditional methods use expert knowledge and often require knowledge of specific system characteristics [1,2]. However, methods based on the use of artificial intelligence [3,4] and fuzzy logic [5,6,7] are increasing. This paper presents the results of a platform that uses vibration data and convolutional neural networks for ball-bearing fault detection. The main novelty of the presented method is that, unlike the classical approaches [8,9,10], it does not use image input data but directly uses a vector of raw data.

The proposed method is for predictive maintenance. This method opposes reactive maintenance, where servicing occurs when a machine breaks down. The reactive maintenance method is associated with a very high risk of failure. Preventive maintenance, on the other hand, although it avoids sudden, unforeseen stoppages and primarily provides greater safety for people in the vicinity, requires more in-depth planning of the entire operation. Additionally, the exact time of failure is unknown, which means that maintenance can be performed even when the machine is not entirely worn out and may have been in operation for a considerable period. With predictive maintenance, on the other hand, it is possible to estimate the remaining operating time of a machine up to failure. Knowing this crucial parameter, one can prepare for service accordingly. In addition, using predictive maintenance, it is possible to estimate the time remaining until the required service and learn about a specific defect in the operation of the machine and the component that should be repaired or replaced.

This paper proposes a model that enables data analysis without using signal processing methods. In order to verify the accuracy of the proposed solution, two other deep learning algorithms were trained for comparison. The first was a pretrained MobileNet-v2 model, and the second one was a multilayer perceptron network. A model based on the MobileNet-v2 architecture performed classification based on direct analysis of the frequency spectrum, in the form of an image. The multilayer perceptron model used data without any signal preprocessing. All models were trained on data collected directly during machine operation. The models allowed the bearing condition to be assigned to one of three states: healthy, outer race damaged, or inner race damaged. This platform enables measurement, data visualization, and fault detection. The use of neural algorithms reduces the impact of noise on the input data and does not require expert knowledge. The classifiers used belong to the deep learning family, so they perform feature extraction independently and, based on the learning set, determine which features of the input data are relevant for fault detection. This is their most significant advantage, as it reduces the impact of noise and increases the efficiency of fault recognition.

## 2. Related Works

Predictive diagnostics for rotating equipment commonly rely on various data sources, including vibration [11,12], acoustics [13,14], vision systems [15,16], and thermal imaging [17,18]. Frequency domain or time–frequency domain analysis approaches, such as fast Fourier transform (FFT), continuous wavelet transform (CWT), and spectrograms obtained through short-time Fourier transform (STFT), are often used for vibration and acoustic analysis. These methods are more prevalent than time-domain analysis, as documented by the authors in [19]. Spectral or time domain data can be analyzed using either probabilistic or neural network approaches. In recent years, probabilistic approaches have become less popular, due to their poor robustness to new or unforeseen parameters, which can lead to false interpretations of the device’s state [20,21]. This is due to the fact that these methods necessitate comprehensive knowledge for developing a reliable system, and real systems are too complex to evaluate using such models. Despite the limitations of each approach, there is a need for a deeper analysis of neural methods.

Recurrent neural networks (RNN) are one of the primary neural network-based methods that use raw data to obtain a model of the object under study [22]. However, the effectiveness of RNNs is highly dependent on the structure of the network, which must be determined each time for new machines and devices, as there is no deterministic way to find the optimal structure. Moreover, RNN is a “shallow” learning method, meaning it does not independently extract hidden features of the system, and the network designer must extract the information hidden in the raw data, requiring significant expertise. Additionally, RNN models are highly susceptible to overtraining.

Support vector machines (SVMs) are commonly used for time, frequency, or time–frequency characteristics analysis, as documented in various studies [23,24,25]. These models achieve a high damage prediction performance and are typically based on image analysis of the frequency or time–frequency spectrum. However, SVM models are known for their significant difficulty with selecting the appropriate activation function, and it is unclear which damage classes a specific kernel function should be used for. Nevertheless, many kernel activation functions have been shown to perform well. The SVM network training time significantly increases when the number of samples is increased during the evaluation period, and the model itself is not easily interpretable. Despite these limitations, SVMs can be valuable for model designers.

In the design of a solution for failure prediction or detection in an industrial environment, the ability of the model to process data from multiple sensors simultaneously is a critical aspect. Often, a system failure is indicated not by just one variable but by a combination of several. To address this, predictive maintenance solution designers adapt their systems to incorporate multiple types of sensor data. Several signal processing techniques, such as ensemble empirical mode decomposition (EEMD), Walsh–Hadamard transform (WHT), and discrete wavelet transform (DWT), have been employed to create a feature base for models, including random forest, support vector machine, artificial neural network, and IBK, based on the fusion of multi-sensor data, as documented in [26]. In addition, designers have used the visualization of raw data from multiple accelerometers in the form of a rectangular matrix and perform classification using a 2D CNN, which is an efficient method for utilizing data from multiple sensors, as stated in [27]. The swarm decomposition (SWD) algorithm with parameters optimized using the BAS algorithm wa employed to segment and pre-process raw data from multiple sensors, as reported in [28]. This optimized neural algorithm enabled sensor fusion-based fault detection.

In many studies on bearing damage detection, the authors tend to overlook the variable rotational speed of the drive. This poses a significant challenge in developing a robust detection algorithm. To address this issue, a solution was proposed in [29]. This approach entails using sparse filtering as a basis for constructing a network for feature extraction, followed by the addition of batch normalization to address the frequency shift and amplitude variation of the measured signal. Finally, a classifier based on softmax regression is created. This method effectively eliminates the effects of speed variation on the performance of the classifier. Another related solution to the challenge of varying operating conditions and adapt diagnostic algorithms to real-world applications is presented in [30]. The proposed hybrid distance-guided adversarial network (HDAN) comprises two parts, namely a convolutional neural network (CNN) used as a feature extractor, and a shared classifier. By utilizing HDAN, a significant improvement in diagnostics over the classical algorithms was observed. In the field of predictive maintenance, the k-nearest neighbors (k-NN) method has been found to be an effective and computationally efficient technique [31,32]. To classify features with this method, it is important to determine the similarity measure between the training and test instances. By selecting an appropriate measure, it is possible to reduce the influence of noise on the classification, but at the same time, this may result in greater blurring of the boundaries between classes. This may affect the effectiveness of the algorithm heuristically and individually when new instances are added. The k-NN method is particularly suitable for the predictive maintenance of rotating equipment, due to its low computational complexity and its ability to operate in unsupervised mode. However, it requires image analysis of the frequency or time–frequency spectrum, rather than the raw data. Other neural methods, including convolutional artificial neural networks (CNN), have been used successfully for predictive maintenance. CNNs are particularly suited for image recognition and object detection tasks, and their popularity has led to the development of new and improved network structures. However, the increasing complexity of these networks often requires more computing power, especially when implemented as “AI on the edge”. Additionally, CNNs are better suited to local field representation, which makes them easier to interpret [33,34]. Nevertheless, CNNs are not as efficient in feature extraction for one-dimensional vector data obtained in raw form from a single sensor, as reported by [35]. In this study, we present a modified CNN network, designed to improve the performance of classification from raw one-dimensional data, as opposed to using time–frequency spectrum image analysis.

## 3. Methodology

During operation, rotating machines emit vibrations with different frequencies, and each component of such a machine emits vibrations with a different amplitude and frequency. Typically, the largest amplitude is characteristic of the vibrations of large components, from the rotational speed of the shaft or other large-mass components. However, with proper analysis, it is also possible to observe the vibrations of small-scale components, which usually have a much smaller amplitude and higher frequency. Knowledge about the vibration acceleration generated during the operation of a machine allows us to obtain an immense amount of information about its condition. One of the observations that can be obtained is information on the condition of roller bearings. These can be found in almost every rotating machine, and their damage is a frequent cause of failures. Owing to the nature of bearings, it is possible to calculate from catalog data the frequencies at which the amplitudes increase when individual components are damaged. In this way, we can diagnose, for example, damage to the outer and inner raceways. When one of these areas is damaged, the rolling elements of the bearing rolling on it cause cyclic beats visible in the frequency spectrum. These are ball pass frequency of the outer race (*BPFO*) (1) and ball pass frequency of the inner race (*BPFI*) (2), respectively [36]. This is caused by the pulse-like signal generated by a rolling element rolling over a damaged rough surface, which excites a wide spectrum of frequencies. This means that the resonant frequencies of specific components, for example bearing components, can be excited. In this way, the damage frequencies of the outer raceway can be observed; they will be visible as individual vertical stripes in the spectrum. For the inner raceway, which rotates at the same frequency as the shaft frequency, the phenomenon of amplitude modulation occurs such that the rolling elements will generate pulses at different distances from the sensor. This will cause the inner race defects to be modulated by the frequency of the rotation of the shaft, and they will be visible in the spectrum as vertical bars with side bands.

(1) $$BPFO=\frac{N_{b}}{2}f_{r}\left(1-\frac{d_{B}}{d_{P}}\cos\theta \right)$$

(2) $$BPFI=\frac{N_{b}}{2}f_{r}\left(1+\frac{d_{B}}{d_{P}}\cos\theta \right)$$

where $N_{b}$ is the number of rolling elements, $d_{B}$ is the diameter of the rolling element, $d_{P}$ is the distance between opposite balls in the bearing, θ the angle of contact, and $f_{r}$ is the shaft rotation frequency. Unfortunately, however, in an industrial environment, accurate measurements are often difficult to perform, and in such cases, it is challenging to analyze the bearing condition. Therefore, it was decided to use neural algorithms to perform such analysis. They allow the extraction of signal features, even with a significant presence of noise and interference. Such solutions may, in future, become very fast to implement and universal. For this research, three sets of bearings were prepared: healthy bearings, and bearings with a damaged inner raceway and outer raceway.

### 3.1. Spectrogram Model

The first model was built to compare traditional solutions based on image data processing. The MobileNet-v2 network structure was used as a base to build the spectrogram model. However, modifications were made to it compared to the version presented in [37]. MobileNet-v2 is a convolutional neural network trained on the ImageNet dataset to classify images into 1000 categories, such as computer, dog, cat, mouse, or human. As a result, it has learned to recognize many features in various images. It was designed to optimize memory usage and other hardware resources, making it ideal for applications running on portable, low-power, and low-memory hardware devices. By using a transfer learning technique, it is possible to take advantage of the fact that the inside of the model is trained to recognize features in images from the ImageNet collection and modify only parts of the model, so that when re-trained, it allows classification into categories other than those originally intended.

(3) ReLU6(x)=min(max(0,x),6)

MobileNet-v2 was built on depth-wise separable convolutions, as efficient building blocks. However, it uses linear bottlenecks between the layers, ensuring that the nonlinearities common in neural network structures do not destroy too much information. Between the bottlenecks are shortcut connections. There are two types of blocks in MobileNet-v2 (Table 1). One are residual blocks with a stride of 1, and the other are downsizing blocks with a stride of 2. Both types of blocks are built with three layers. The first is a 1 × 1 convolution with the activation function *ReLU*6 (3), the second is a depth-wise convolution, and the third is another convolution, but this time without applying any nonlinearity. It is claimed that if *ReLU* is reused, the deep networks only have the power of a linear classifier on the non-zero volume part of the output domain.

The network structure used for fault detection can be seen in (Table 2). The dimensions of the input layer have been changed to 160 × 160 instead of 224 × 224, which reduces the number of model parameters and speeds up the algorithm. After the last layer, whose dimensions are equal to $5^{2}\times 1280$, to reduce dimensionality, the GlobalAvaragePooling2D layer was used. The next layer is a dropout with a parameter of 0.2, this makes 20% of all values zeroed during network training, and the rest are unchanged. This is a known method for preventing overfitting. Next, a fully connected layer was added to classify into three designated classes. The last layer is softmax, and its action makes the sum of outputs of all neurons sum to 1. This solution allows us to obtain the confidence of assignment to a given class.

The final model consisted of 2,261,827 parameters, of which only 3843 were subject to optimization during learning (Table 3). Parameters that were not subject to optimization already had optimized values for proper feature extraction from images, thanks to previous training on the ImageNet set. This method of operation allows significantly reducing the learning time of the algorithm compared to the case of learning the whole model.

### 3.2. Time Series Model

An alternative method of spectrogram processing and classification was also proposed; that is, the use of raw measurements from the sensor, without the use of signal processing methods such as Fourier transform, wavelet transform, or spectrogram generation. In the considered case, the so-called time series were analyzed [38]. For this purpose, the model structure proposed in [39] was used, where a fully connected neural network acts as a feature extractor. This structure uses three basic convolutional blocks. Each of them consists of a successive convolution layer, a normalization layer, and an activation function, which in this case is *ReLU*. The convolution operation is implemented using three kernels of size {3,3,3} without striding, each of which also has 64 filters. The structure of the basic convolution block [39] is expressed by the Equations (4)–(6).

**Figure 1:**
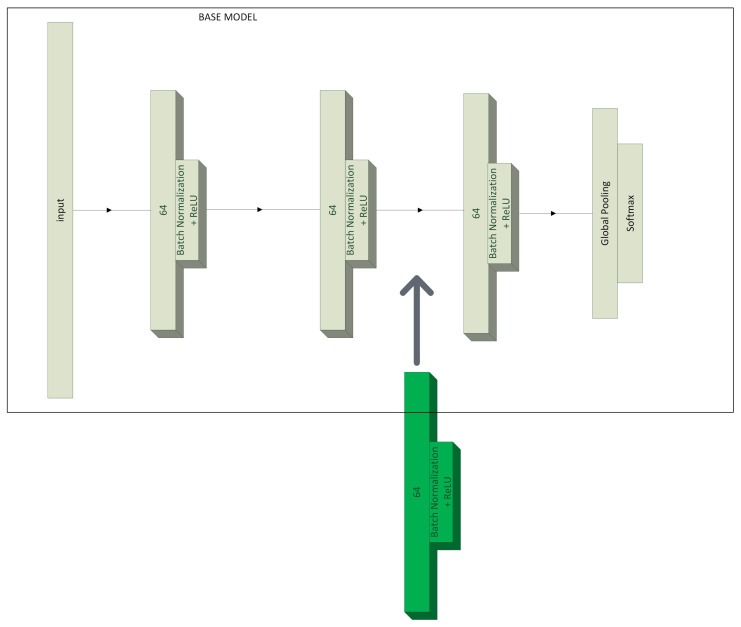
Proposed time series model modification.

**Figure 2:**
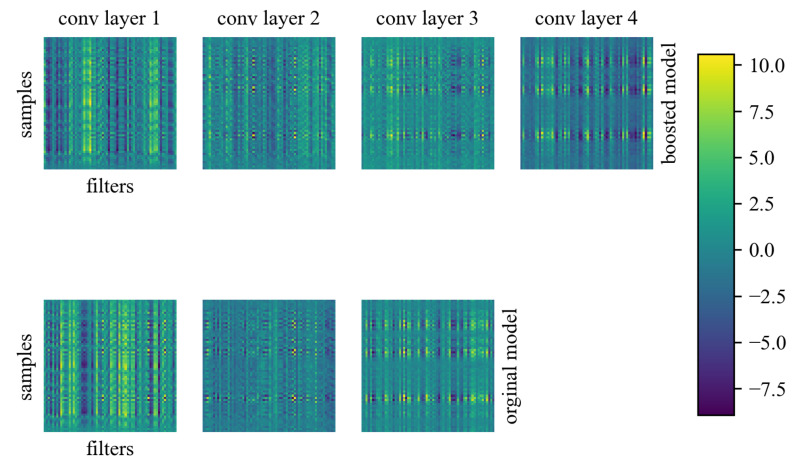
Feature maps for the originally proposed model and boosted algorithm. Each map consists of 64 samples from 11,025 long vectors on the y-axis and 64 filters on the x-axis.

(4) y=W⊗x+b

(5) $$s=BN\left(y\right)$$

(6) $$h=ReLU\left(s\right)$$

where ⊗ is the convolution operator, W is the vector of weights, b is the bias, and *x* is the vector of inputs. In the original version, the architecture consisted of three convolutional blocks arranged sequentially from the input. However, when an additional convolutional block (Figure 1) was added, a remarkably shorter learning time and more than double the validation loss was achieved. The additional block also had no significant effect on the execution time of the algorithm, which on average increased by 9.54 ms. In convolutional blocks, no merging operation was used; a similar strategy as adopted in ResNet [40]. The output block consists of a GlobalAvaragePooling1D layer and a fully connected layer with a softmax activation function. The pooling layer generates a feature map for every class, computes the average of each map, and feeds the result into the softmax layer. The use of the softmax function as an activation function in the final layer, similarly to the model based on spectrogram classification, allows for a sum of the output neuron values equal to 1, which can be directly translated into the classification confidence for each class. After preliminary tests, it was decided to only implement the modified algorithm. An additional convolutional block allowed, at a low computational cost, improving the feature extraction from a noisy signal. Finally, the model consisted of 38,531 parameters in total. Only 512 of them were not optimized during training (Table 3). In Figure 2, there are feature maps for both models. On the y-axis, there is a fragment of 64 samples from a vector of 11,025 samples, while on the x-axis, there are 64 filter variations. The brighter colors correspond to higher values, while the darker colors correspond to lower values. Thanks to this, it is possible to observe how the activation was distributed in the model structure. In the case of the original model containing three convolutional blocks, the algorithm dynamically adopted higher values in feature maps (specific very bright points). By deepening the structure of the network with an additional convolutional block, we obtained an algorithm that reacted less aggressively and thus could perform even better as a preprocessor of batch data, by marginalizing noise and including only relevant features.

### 3.3. Multilayer Perceptron Model

**Figure 3:**
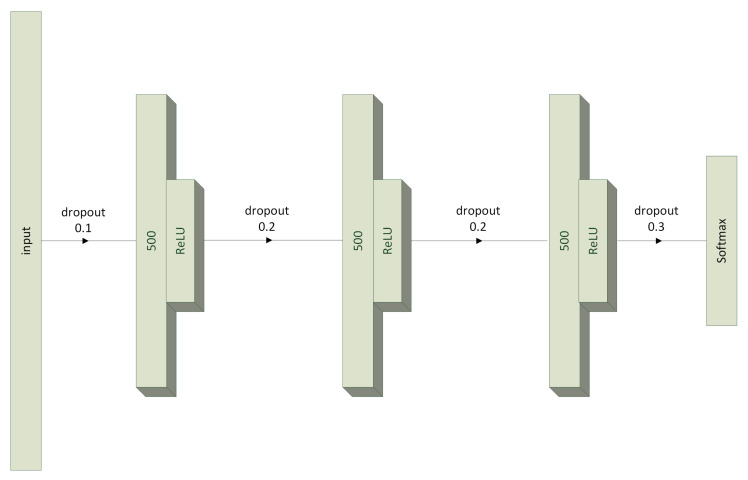
Architecture of the MLP model.

In order to compare with other popular neural algorithms, we attempted to train one more model. This was a classical multilayer perceptron (MLP) network with the structure proposed in [41]. It consisted of three hidden layers (8) containing 500 neurons, with each layer preceded by a dropout layer (7) with values of {0.1,0.2,0.2} and ending with a *ReLU* activation function (9). The last layer of the network was again softmax, this time preceded by a dropout layer with a value of 0.3. The entire network structure can be traced in Figure 3.

(7) $$\tilde{x}=f_{dropout}\left(x\right)$$

(8) $$y=W\cdot \tilde{x}+b$$

(9) $$h=ReLU\left(y\right)$$

It is the presence of nonlinearity in the form of the *ReLU* activation function that is supposed to prevent gradient saturation of a deep network. On the other hand, dropout was used to prevent the overfitting phenomenon. The final MLP model consisted of 6,015,503 parameters (Table 3), all of which are subject to optimization.

### 3.4. Method Framework

**Figure 4:**
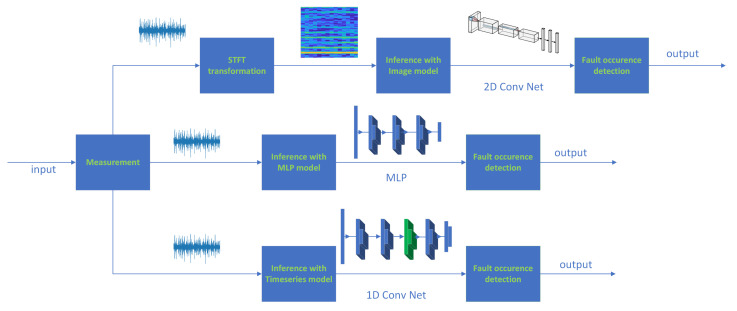
Method framework diagram.

Three methods were proposed to implement the detection algorithm based on deep learning. Two of them were based on time series data processing. The first was the proposed boosted 1D CNN algorithm, and the second was an MLP network. Both performed inference based on the data directly from the sensor, without using any signal processing methods. Another was a traditional method using an algorithm based on image processing, specifically signal spectrograms. The frameworks of all three algorithms are presented. In Figure 4, and we can see a data flow diagram for each algorithm.

#### 3.4.1. Framework for MLP and 1D CNN Time Series Models

**Figure 5:**
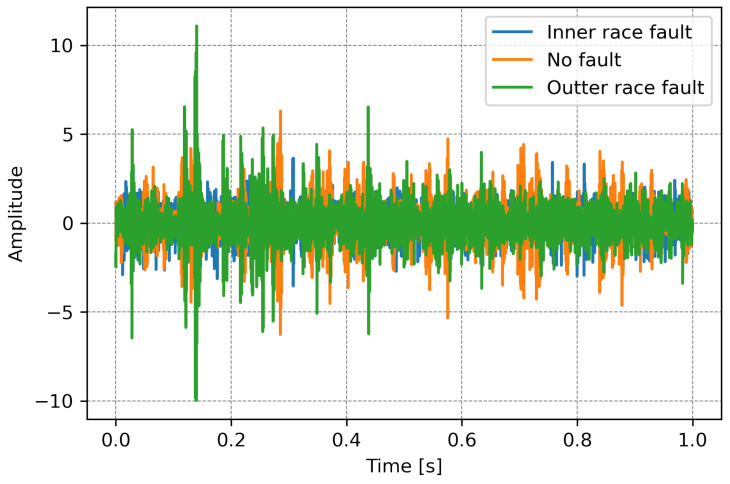
“Raw” data in the time domain.

The procedure for the first two algorithms based on analyzing data directly from the sensor was identical. The measured acceleration values are stored and analyzed as raw data (Figure 5). The neural network makes an inference based on the sensor’s direct measurements. This is possible because deep learning algorithms can extract features independently from the data analyzed during learning. This results in a black-box model, i.e., the algorithm’s designer does not know the exact rules of its operation because they are extracted directly from the data provided by the system designer during learning.

Algorithms are designed to analyze data in a one-dimensional vector, although the models are prepared in a way that allows them to fit the dimensions for analyzing a multi-channel input. For example, if a three-axis accelerometer is used, two additional channels can be added easily, the same as any other set of sensors. The models perform inference based on the three-channel input, using operations for every channel [38]. During operation, one second of acceleration data are recorded and saved as a .wav file. Then algorithm reads the data as a 1D vector and performs inference based on this data. The output is the confidence of classification for each class. Before entering the model, the data are standardized using a standardized z-score (11). This approach significantly shortens the computational trajectory and speeds up the computation, due to eliminating signal processing.

#### 3.4.2. Framework for the Spectrogram Model

**Figure 6:**
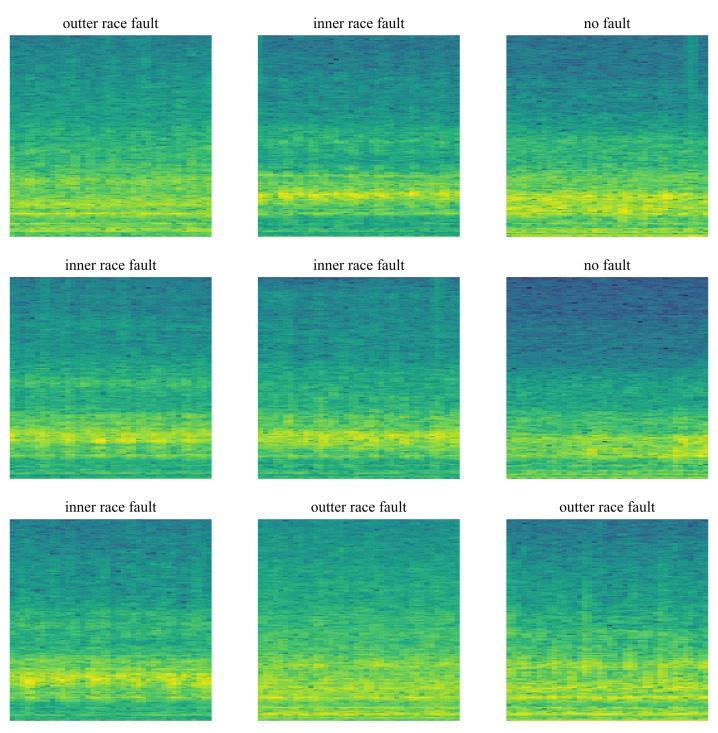
Sample spectrogram images for each class.

The framework for the algorithm based on image analysis is presented as follows: First, the acceleration is measured using an acceleration sensor. Then the data are analyzed using a processing unit. It records one second of measurement, with a sampling rate of 11,025 Hz. After recording, the data are standardized according to the z-score standardization mentioned in (11). The next step is to process the signal using a short-time Fourier transform (STFT) and to visualize the results as a spectrogram (Figure 6). The generated image is stored without axes and their description, only the core of the spectrogram image. The data prepared in this way go to the input of the neural model, which performs inference and outputs the classification result. The procedure for preparing the algorithm’s batch data will be presented in detail below.

A dataset consisting of spectrograms of the measured signal was created using known signal analysis methods. One spectrogram image represents one second of measurement. In the case of signal processing using the Fourier transform, the result is a frequency spectrum, which contains only information about the relationship between the frequency of the signal and its amplitude. Information about what happened to the signal over time disappears. The influence of time can be taken into account using a spectrogram, where the horizontal axis represents time, the vertical axis represents frequency, and the intensity of coloring reflects the amplitude of a particular frequency at a given time interval. The spectrogram is a better solution due to the nature of convolutional neural networks. It has more features that are easily recognizable to the neural network, such as colors, saturation, transitions between colors, and brightness. In Figure 6, a few sample spectrogram images from the dataset are shown. Images were saved without axes, to provide only relevant information in terms of the CNN algorithm. The spectrogram of a signal is obtained by visualizing the result of computing multiple Fourier transforms for very short time durations. This algorithm is called the time-dependent Fourier transform or the short-time Fourier transform (STFT) [42]. For a signal x[n], this is defined as (10)

(10) $$X\left[n,\lambda \right)=\Sigma_{\left(m=-\infty \right)}^{\infty }x\left[n+m\right]w\left[m\right]e^{-j\lambda m}$$

where w[n] is a window in the data sequence. In the case of STFT, the one-dimensional sequence x[n] becomes a two-dimensional function from the time variable *n*, which is a discrete variable, and the frequency λ, which is continuous. The above equation can also be interpreted as the Fourier transform of the offset signal x[n+m], with a window w[m].

The considered window is stationary, and then the signal is shifted through the window, so for each value of *n*, a different part of the signal is analyzed. A similar algorithm is used in practice, but the signal under analysis is not shifted by one sample but by a more significant value. It is often given as a percentage value of the overlapping of successively analyzed signal windows. Such an operation has many advantages. First, it reduces edge effects, distortions in the extreme areas of the window resulting from the multiplication of sample amplitudes by values of the window function close to zero. Second, this increases the time resolution of the analysis. The processed signal can be visualized with a color intensity map, producing a spectrogram. A Hamming window with a width of 1024 samples and an overlap of 75% was used when processing the accelerometer signals. The sampling frequency was 11,025 Hz, resulting in a frequency resolution of the transform of 5.38 Hz.

## 4. Measurement Stand

The accelerometer used was a digital piezoelectric acceleration sensor equipped with a built-in transducer and USB interface. This was contained in a robust, hermetically sealed stainless steel housing, protecting it from the harmful effects of the environment in which it operates. This solution makes it possible to measure vibration acceleration without bulky and inconvenient measuring cards, transducers, and conditioners. Such a solution makes the platform extremely practical. The accelerometer sends a data stream, using the audio format, which is then read by a minicomputer. The software developed allows the measurement file to be saved in .wav format. It is then possible to present the measured accelerations in a window application displayed on an external screen. It displays the signal in the time domain, frequency domain, and as a spectrogram. This solution allows connecting the sensor directly to the computing unit and using readily available, popular minicomputers equipped with a USB connector, such as the Beagle Board, Intel NUC, Raspberry Pi, and Latte Panda, NVIDIA Jetson. It is very convenient to develop custom software and read data from the sensor. It also uses an interface used in popular audio devices (e.g., microphones) to communicate with the computing unit. The multitude of available libraries, available in many programming languages (from C++ through LabView to C# and Python) allows the creation of stand-alone applications using the chosen accelerometer. This solution was chosen because the application was implemented directly for real-time operation. The sensor has the following:

- linear (+/− 5%) frequency response from 2 to 8000 Hz,
- a transducer with a resolution of 16 or 24 bits,
- measurement range:
  – channel A +/− 196 m/s^2^
  – channel B +/− 98 m/s^2^
- sensitivity:
  – channel A 4% FSV/g (Full Scale Value)
  – channel B 7.96% FSV/g

**Figure 7:**
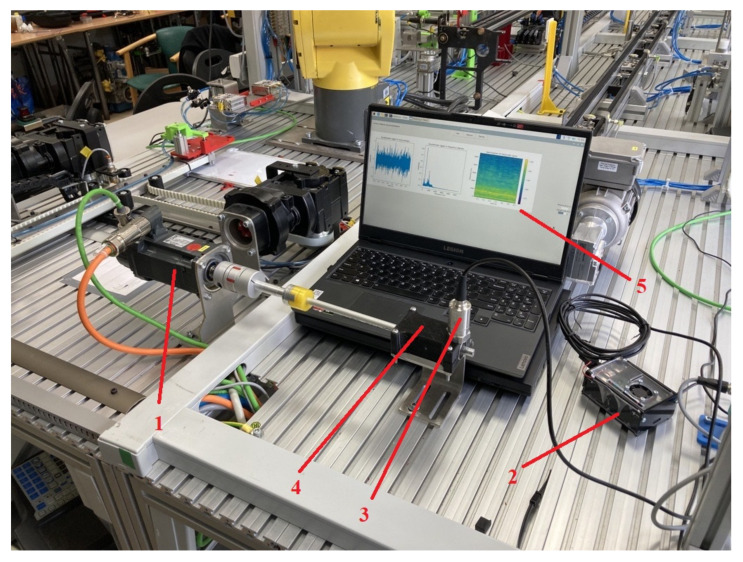
Test rig and device with a notebook as a display. 1—servo motor, 2—minicomputer, 3—accelerometer, 4—bearing housing, 5—display.

A Raspberry PI 4B minicomputer was used as the computing unit. It has a Quad-core 64-bit ARM-Cortex A72 processor running at 1.5 GHz and 4 Gigabyte LPDDR4 RAM. Such components allow the detection model to run in real time, in parallel with the GUI application. This solution also has mobility and compactness (Figure 7).

**Figure 8:**
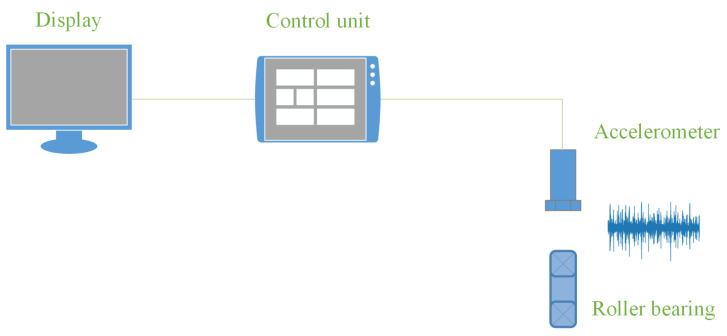
Device diagram.

This application also enables the user to select measurement parameters: the duration, sampling frequency, and recording resolution (16 and 24 bits). Based on the measurements and the trained deep learning model, the device informs the user about the current state of the tested bearing. It also displays the confidence with which the algorithm decides the state of the tested element and the execution time of the detection algorithm. A schematic of the device can be seen in Figure 8.

**Figure 9:**
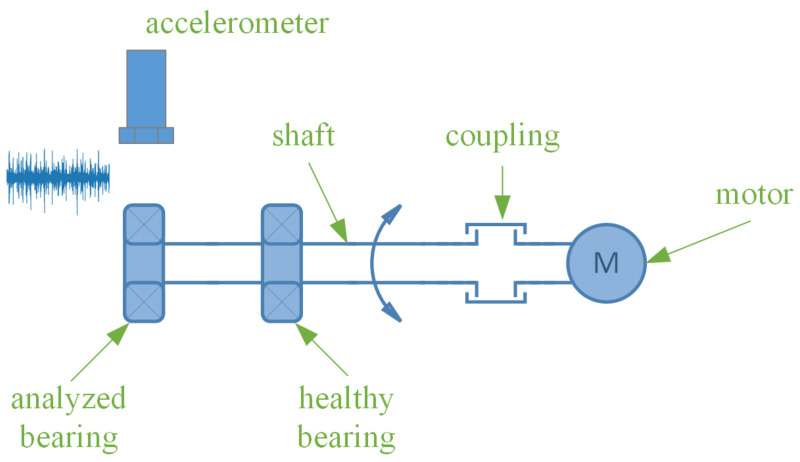
Test rig diagram.

In order to collect the data necessary to learn the model, a test rig consisting of a bearing-mounted shaft driven by an electric motor was build. The shaft is coupled to the electric motor at one end and supported by two bearings at the other end. The accelerometer is mounted in the bearing housing, so that measurements can be taken for the bearing furthest away from the drive (Figure 9). It is mounted perpendicular to the shaft axis, making it possible to measure vibration accelerations in the perpendicular plane. In the case studied, the shaft did not carry any additional loads. The electric motor used was a servo-type drive. This was chosen because of its very high dynamics and following constant speed. A closed control loop and very low hysteresis allowed for precise setting and maintenance of the rotational speed.

## 5. Data Acquisition

**Figure 10:**
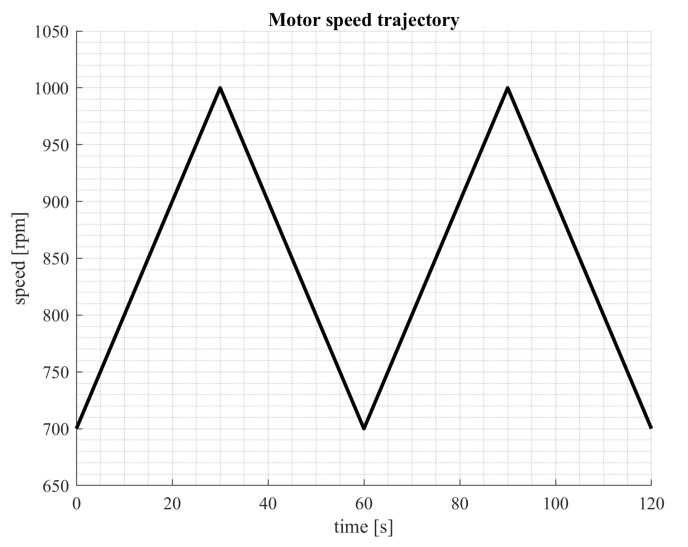
Motor speed trajectory.

Using a PLC controller, a sequence was programmed in which the electric motor accelerated from 700 rpm to 1000 rpm in 30 s, and then decelerated from 1000 rpm to 700 rpm (Figure 10) in the same time interval. It was decided to analyze a variable range of speeds, rather than a constant speed, because this is more difficult to accomplish with standard expert-based methods. The frequencies calculated from the (1) and (2) equations are dependent on the shaft speed. Adopting a variable speed range also allows the algorithm to get closer to the operation of a real object and has tremendous implementation potential. In order to collect data for learning algorithms, a measurement of 20 min was performed for each bearing state. There is no specific set of rules to determine the size of a training dataset. However, it is said that for the classification problem, there should be at least 1000 data samples for each class when a designer tries to use a non-pretrained model.The measurement length was chosen based on this rule and heuristically. During the measurements, the programmed trajectory of the motor speed change was executed cyclically. Finally, about 60 min of vibration acceleration measurements were obtained.

The algorithms used required different input data, so preparing two separate learning datasets was necessary. However, despite the difference in the data type, samples of 1 s were analyzed in both cases, resulting in a set size of 3600 samples, 1200 for each of the analyzed states. However, in both cases, the data were subjected to z-score standardization, which measures the distance of a data point from the mean using the standard deviation. The normalized data set had a mean of 0 and a standard deviation of 1, and retained the shape properties of the original data set (same skewness and kurtosis). This type of normalization is common in time series classification problems, see [41]. For sampled data, *x* whose mean is $\bar{X}$, and whose standard deviation is *S*, the *z*-score for a data point was calculated according to the Equation (11).

(11) $$z=\frac{x-\bar{X}}{S}$$

Due to the specific operation of the measurement system, each of the measurements was recorded in a single .wav file, so it was necessary to divide the data into fragments of a given length. Finally for MLP and 1D CNN models, a .csv file was created, containing in each line, 11,025 signal samples (this corresponds to a measurement of one second in length). For the spectrogram model, set of 3600 spectrogram images was used. The data prepared in this way made it possible to use them while training the algorithms.

## 6. Training Results

**Figure 11:**
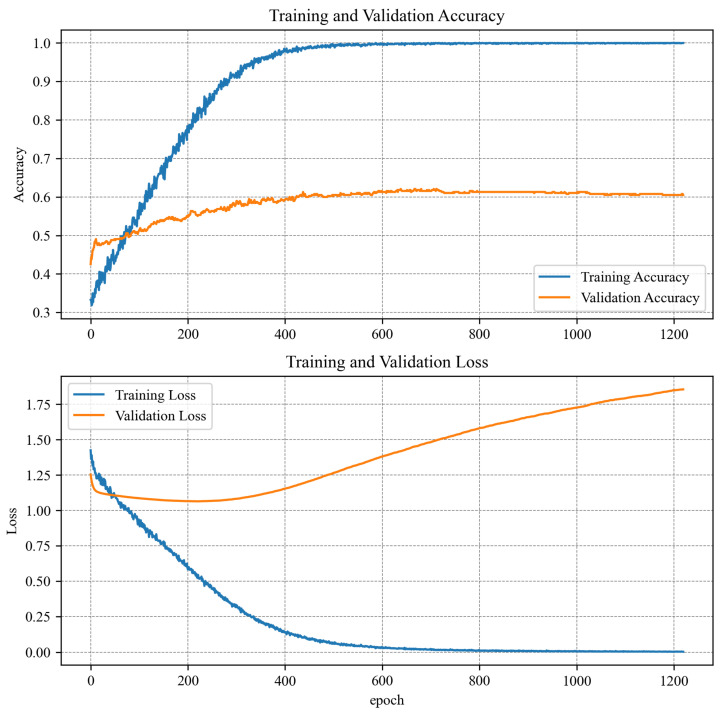
Training results for the MLP model.

**Figure 12:**
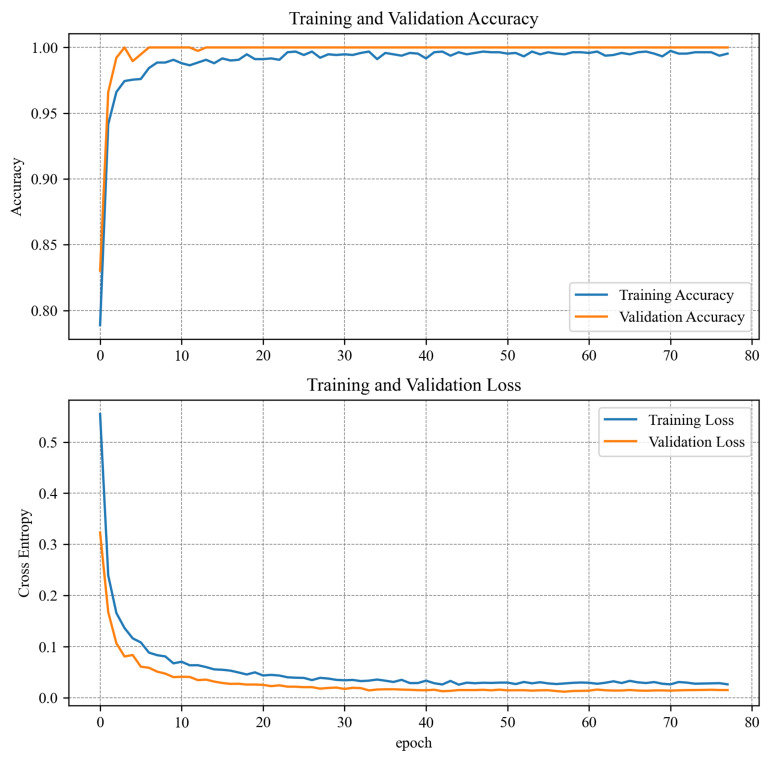
Training results for the spectrogram model.

**Figure 13:**
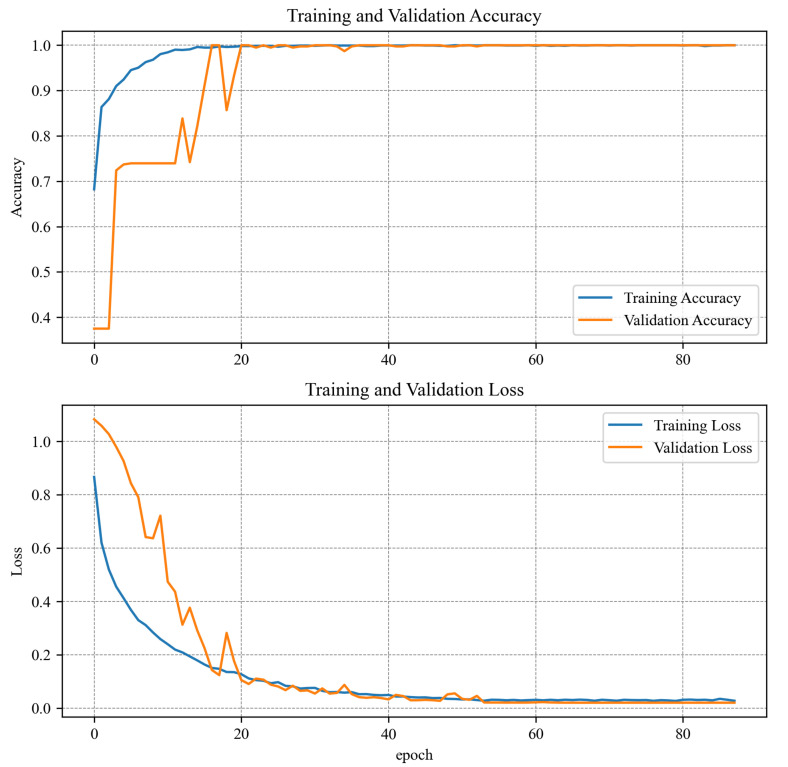
Training results for the time series model.

Spectrogram-based and time series 1D CNN algorithms were trained with the same options. The data set was divided into learning, test, and validation sets. Different splits were tested, and the best results were obtained when 80% of all data were allocated to the learning set and 10% to the test and validation sets. Such a split maintained a high learning rate (higher than the 90:5:5% split) and a high variance for the training and test or validation data. Due to the small amount of learning data, a batch size of 32 was used in both cases. The stochastic gradient descent (SGD) algorithm was chosen as an optimizer because it provided sufficient precision and learning time. The learning rate was actively reduced from 0.01 to 0.000001 if there was no change in the validation loss value for five epochs. In addition to actively changing the learning rate, an automatic learning stop was also used. This time the stoppage condition was that there was no improvement in validation loss for 20 epochs. Thus, the spectrogram model was stopped after 78 epochs and the time series model after 88 epochs. For the algorithm based on the MLP network, different learning options, splits, and optimizers (Stochastic Gradient Descent, ADAM and Adadelta) were tested, but it was impossible to fit the algorithm with a higher precision than 60% for validation and testing data sets. The algorithm was overfitting rapidly. Probably, more training data could have increased the performance a bit. Even though the algorithm was trained for over 1000 epochs, none of the tested optimizers could manage to find the minimum again.The training results with the Adadelta optimizer can be found in Figure 11. A comparison of the learning results of all algorithms can be seen in Table 4. The graphs in Figure 12 and Figure 13 show how spectrogram and time series model performed during training. It is worth noting that the spectrogram model, which had been pretrained, reached satisfactory values very quickly. Model analyzing raw data were trained from scratch and needed more time to reach satisfactory results. Due to the poor learning results for the MLP algorithm, its further implementation on the target device was abandoned.

## 7. Testing Results

**Figure 14:**
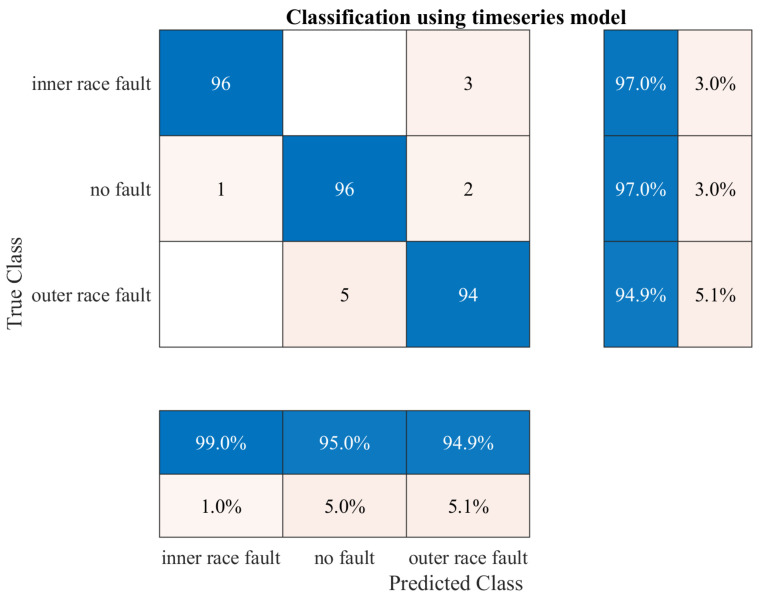
Confusion matrix for the time series model.

**Figure 15:**
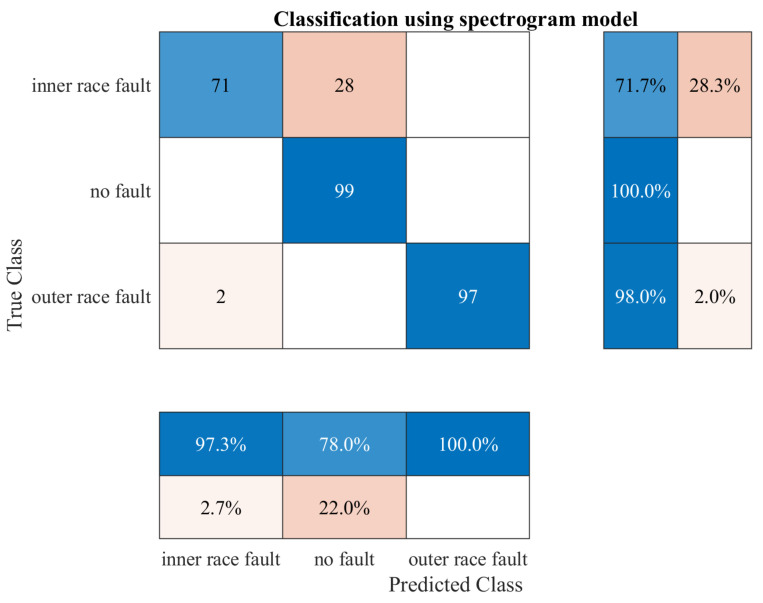
Confusion matrix for the spectrogram model.

The trained and implemented models were tested on a laboratory bench. A script was created to record the algorithm inference results, classification confidence, and algorithm execution time. Such data allowed comparing the performance of the two models, not only in terms of accuracy of performance, but also inference time. Good performance was essential, because the algorithms ran on a low-power minicomputer, so the execution time significantly affected the performance of the entire detection system. However, the most critical indicator in failure detection is the precision of the detection algorithm. Confusion matrices were used to compare the performance of the algorithms. A confusion matrix is an N × N matrix used to evaluate a classification model’s performance, where N is the number of target classes. The matrix compares the actual target values with those predicted by the deep learning model. This gave us a holistic view of how well our classification model performed and what errors it made. In the case study, the model based on time series actions performed much better in the tests, because it achieved a 96.3% precision during the real-time tests (Figure 14). The second model based on image analysis performed worse in the tests, achieving an average precision of 90.6% (Figure 15). The most concerning factor was that the spectrogram model misclassified many inner race faults as a healthy bearing. This could cause a dangerous situation in a real detection system. The spectrogram model performed very well for the two classes of no fault and outer race fault, while for the third, the precision was significantly lower. It is possible that the precision of the model could be improved by expanding the learning dataset. A model based on image analysis is much more demanding to train and needs more data, despite being pretrained, which may have proved crucial in this case. In conclusion, the time series model performed much better during the tests.

**Figure 16:**
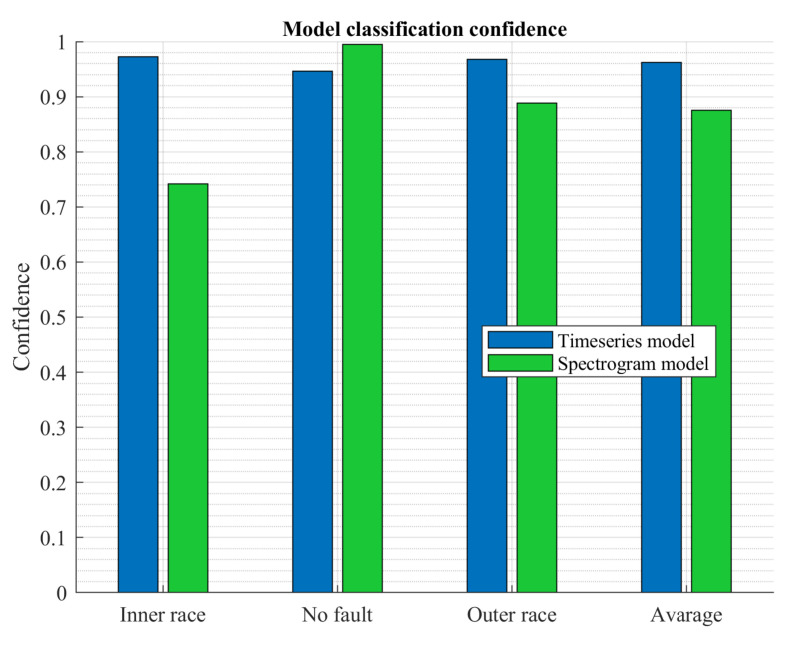
Confidence test results.

**Figure 17:**
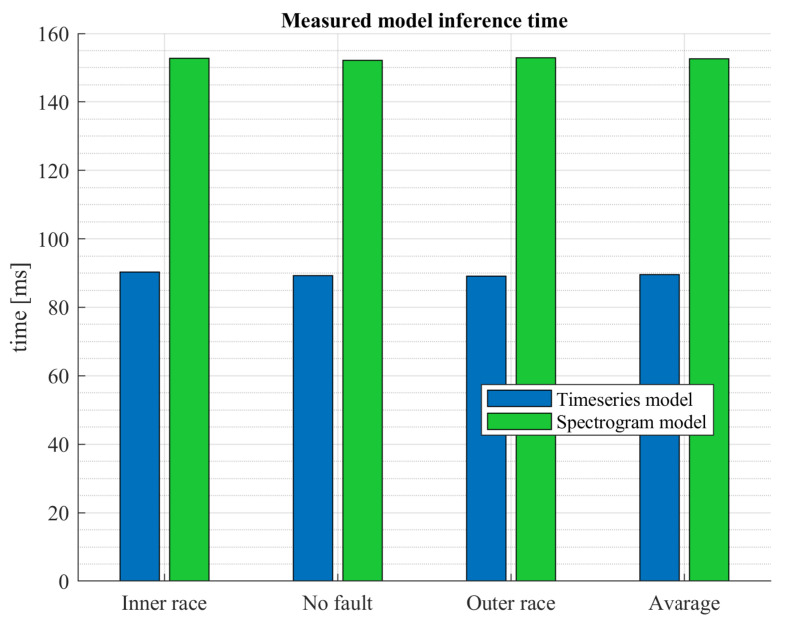
Inference time test results.

Figure 16 shows a comparison of classification confidence for both algorithms. The graph shows the average confidence value for each class. It can be seen that the time series model performed the classification with a significantly higher average confidence, around 95%, which is a satisfactory result. In contrast, interestingly, for the spectrogram-based model, the confidence was significantly lower, and the average confidence was 87%. It was close to 100% for the no fault class but significantly lower for the inner race fault class (74%). The execution time of both algorithms can also be compared (Figure 17). Here the time series based algorithm executed in an average time of 89 ms. The inference was much shorter than the image classifier model, where the average time was 153 ms. The time series model executed over 11 times in one second, while the spectrogram model executed over 6.5 times. This difference is significant. No large differences were observed between the classification times for each class.

## 8. Conclusions

A new method for fault detection in rotating machines is proposed in this research work. This method is based on deep learning neural networks. This work presented not only a new method of predictive maintenance but also proposed a modification of convolutional networks to significantly improve the network learning process itself. The proposed method is not based on feature extraction of image data, as in conventional 2D convolutional networks, but on automatically finding a set of features from raw 1D data. Such a solution reduces the computational cost of algorithm execution and improves the accuracy of failure prediction compared to the classical methods:

- average algorithm execution time reduced by 42% (64 ms)
- avarage precision during real time tests increased by 5.7%
- avarage confidence during real time tests increased by 8%

Adding a convolutional block allowed us to increase the preprocessing properties of the algorithm at a negligible computational cost. It has also been shown that, unlike classical deep neural algorithms (MLP), the proposed algorithm performs well with data that contains measurement noise. It can be concluded that it plays the role of both feature extractor and preprocessor, which made it possible to achieve such satisfactory classification results.

Based on the experimental results and performance evaluation of the learning algorithms, it was observed that the 1D CNN model used in this study outperformed the traditional method of spectrogram analysis in terms of inference speed, accuracy, and confidence. Additionally, the proposed 1D CNN model has the advantage of not requiring signal processing and preprocessing techniques, resulting in computational savings.

The modified inference network structure was found to be more robust to noise in the input signal and false input data. The additional convolutional layer in the proposed solution helps to minimize the influence of such data. Furthermore, the proposed solution is able to handle shaft speed fluctuations effectively, resulting in an improved efficiency.

All the data used in the study came from real objects, which further increase the advantages of the proposed solution. The proposed solution is also suitable for low-power, low-memory devices in AI-on-the-edge applications.

In conclusion, the proposed methods and modifications make this solution ideal for predictive maintenance strategies. Additionally, the system could be extended with other predictive algorithms in the future, to achieve a more comprehensive device.

## Figures and Tables

**Table 1:** Bottleneck residual block transforming [37] from *k* to *k*′ channels, with stride *s*, and expansion factor *t*.

Input	Operator	Output
h×w×tk	3 × 3 dwise s = s, ReLU6	$\frac{h}{s}\times \frac{w}{s}\times \left(tk\right)$
h×w×k	1 × 1 conv2d, ReLU6	h×w×(tk)
$\frac{h}{s}\times \frac{w}{s}\times tk$	linear 1 × 1, conv2d	$\frac{h}{s}\times \frac{w}{s}\times k^{\prime }$

*h* - height, *w* - width.

**Table 2:** Spectrogram model structure.

Operator	Output
input layer	$160^{2}\times \;3$
MobileNet-v2 body	
GlobalAveragePooling2D	1280×1
Dropout	1280×1
Dense	3×1
Softmax	3×1

**Table 3:** Spectrogram model parameters.

Model	Non-Trainable	Trainable	Total
Spectrogram	2,257,984	3843	2,261,827
4 conv blocks	512	38,019	38,531
3 conv blocks	384	25,539	25,923
MLP	0	6,015,503	6,015,503

**Table 4:** Model training results.

Parameter	Spectrogram	Time Series CNN	Time Series MLP
Training loss	0.0278	0.0279	0.0028
Training accuracy	96.43%	97.26%	99.93%
Validation loss	0.0144	0.0206	1.8550
Validation accuracy	97.76%	99.32%	60.52%
Epochs	78	88	1220

## Data Availability

The data presented in this study are available on request from the corresponding author.

## Abbreviations

The following abbreviations are used in this manuscript:

FFT	Fast Fourier Transform
CWT	Continuous Wavelet Transform
STFT	Short-Time Fourier Transform
RNN	Recurrent Neural Networks
SVM	Support Vector Machines
EEMD	Ensemble Empirical Mode Decomposition
WHT	Walsh-Hadamard Transform
DWT	Discrete Wavelet Transform
CNN	Convolutional Neural Network
SWD	Swarm Decomposition
BAS	Beetle Antennae Search
HDAN	hybrid distance-guided adversarial network
k-NN	k-Nearest Neighbours
AI	Artificial Intelligence
BPFO	Ball Pass Frequency of the Outer race
BPFI	Ball Pass Frequency of the Inner race
RELU	Rectified Linear Unit
MLP	Multilayer Perceptron
PLC	Programmable Logic Controller
SGD	Stochastic Gradient Descent
ADAM	Adaptive Moment Estimation
1D	One-dimensional
2D	Two-dimensional
